# A simple *in vitro* tumor chemosensitivity assay based on cell penetrating peptide tagged luciferase

**DOI:** 10.1371/journal.pone.0186184

**Published:** 2017-11-10

**Authors:** Tingyu Yu, Jiao Lin, Jin Zhao, Wei Huang, Lingwen Zeng, Zhiyuan Fang, Ning Xu

**Affiliations:** 1 Second Clinical Medical College, Guangzhou University of Chinese Medicine, Guangzhou, China; 2 Department of Clinical Laboratory, Guangdong Provincial Hospital of Chinese Medicine (The Second Affiliated Hospital of Guangzhou University of Chinese Medicine), Guangzhou, China; 3 Key Laboratory of Regenerative Biology, South China Institute for Stem Cell Biology and Regenerative Medicine, Guangzhou Institutes of Biomedicine and Health, Chinese Academy of Sciences, Guangzhou, China; 4 Tissue Engineering and Stem Cells Research Center, Department of Immunology, Guizhou Medical University, Guiyang, China; Advanced Centre for Treatment Research and Education in Cancer, INDIA

## Abstract

The analysis of intracellular ATP can reveal the response of cells to different treatments and is important for individualized medicine. In the present study, we developed a cell penetrating peptides (CPPs) tagged luciferase (TAT-LUC) for tumor chemosensitivity assay. The activity of recombinant TAT-LUC was evaluated using ATP standard solution and tumor cells. This recombinant TAT-LUC was then used for the analysis of sensitivity index (SI) of four strains of tumor cells. The results showed that TAT-LUC could detect less than 10 nM extracellular ATP with a strong correlation between the luminescence intensity and the ATP content (R^2^ = 0.994). Without cell lysis, the detection limit for intracellular ATP analysis was 40 tumor cells. Furthermore, chemosensitivity of four strains of tumor cells (Skov-3/DDP, A549/DDP, MDA-MB-231, Huh-7) was determined by this assay successfully. The cell penetration ability of TAT-LUC enables the assay not only to reflect drug resistance of tumor cells real-timely but also to minimize the test time, which can be a valuable aid for personalized cancer chemotherapy.

## Introduction

The determination of tumor sensitivity can bring great benefits for cancer patients. Due to the prosperity of precision medicine, much attention has been attracted by tumor chemosensitivity assay guided personalized therapy in recent years[1]. A large number of clinical studies have shown that *in vitro* tumor chemosensitivity assay has positive correlation with clinical outcomes[2, 3].

There are several methods for tumor chemosensitivity testing, including the histoculture drug response assay (HDRA), collagen gel droplet embedded culture drug sensitivity test (CD-DST), succinate dehydrogenase inhibition (SDI) test, MTT assay, differential staining cytotoxicity (DISC) assays, colony formation assays, flow cytometry and adenosine triphosphate-tumor chemosensitivity assay (ATP-TCA), *etc*[4–9]. The ATP-TCA is a reliable method that uses intracellular production of ATP as the indicator of cell viability[10], which is widely used for determining drug sensitivity of solid tumors [11, 12].

**Fig 1:**
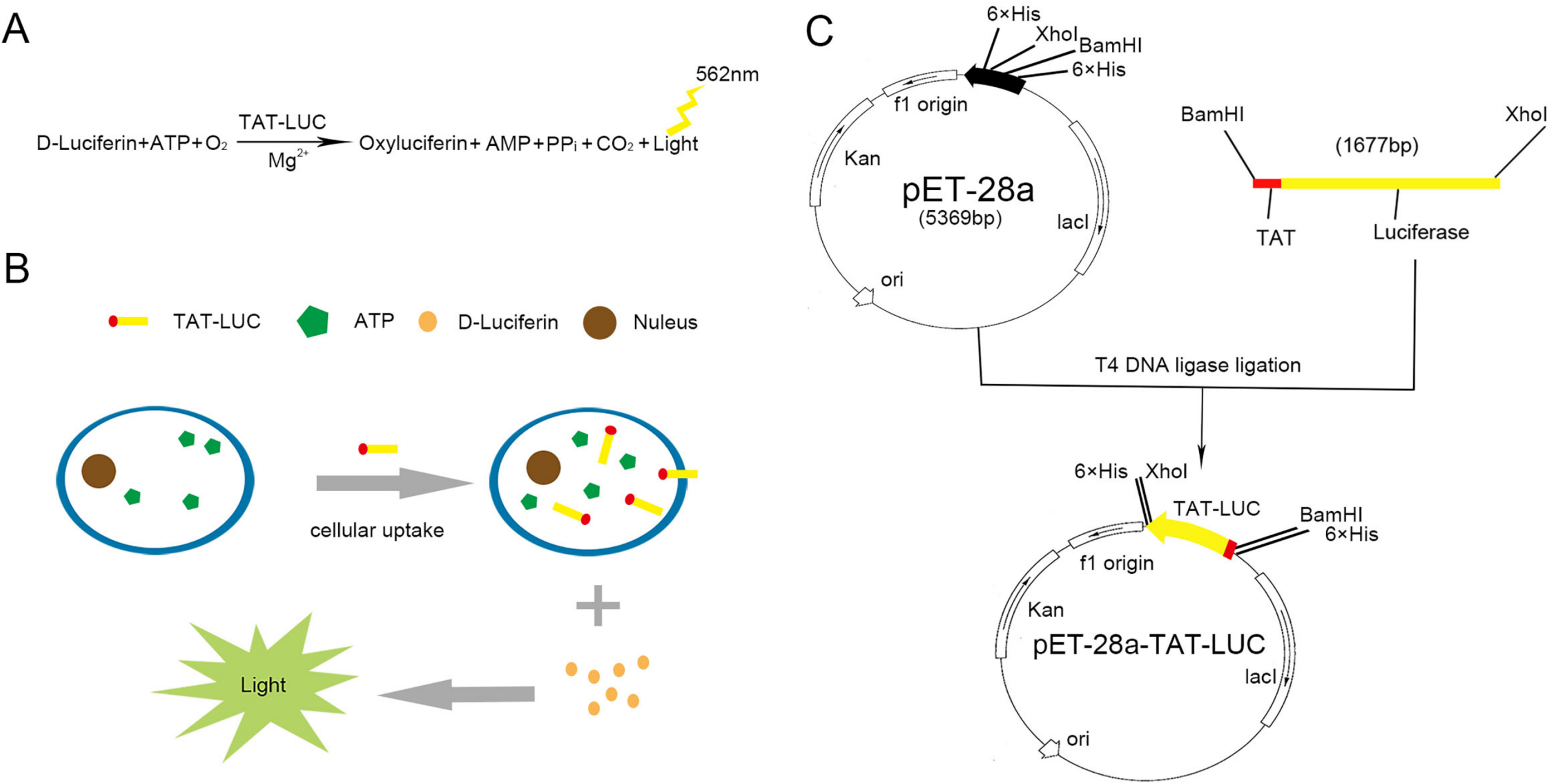
Schematic illustration of the TAT-LUC for intracellular ATP detection. (A) Bioluminescent method based on the luciferin-luciferase reaction (emission wavelength 562 nm). (B) TAT-LUC penetrates cell membrane and ATP powers the luciferase-mediated luminescence production. (C) Construction of the expression plasmid pET-28a-TAT-LUC. TAT-LUC was cloned into the multiple cloning site of the pET-28a vector using *Xho* I and *Bam*H I as indicated. Lac I, the lac repressor; Kan, kanamycin resistance gene; ori, origin of replication.

ATP is the basic energy unit of cells that is rapidly hydrolyzed after cell death. However, there is a dynamic equilibrium between the hydrolysis and regeneration of ATP, which keeps the intracellular content of ATP basically unchanged[13]. Detection of ATP can be used for cell activity analysis, cytotoxic screening and cell proliferation studies[14, 15]. Among the several methods for ATP measuring, bioluminescent method based on the luciferin-luciferase reaction is the most popular one due to its high sensitivity and reliability[16, 17]. In this reaction, luciferase can catalyze the oxidation of luciferin, resulting the releasing of luminescence under aerobic conditions (Fig 1A). At the same time, ATP was converted to AMP and the luminescence intensity was positively correlated with ATP content and the number of living cells[18, 19]. This assay consists of two processes: lysing of cells to extract ATP, and reaction of extracted ATP with luciferin-luciferase. However, there still have few problems in this assay. Above all, ATP can be hydrolyzed by the cell-releasing ATPases before reacting with luciferase[20, 21]. Besides, the concentration difference of luciferin-luciferase between tests will affect the final bioluminescence intensity.

For precise measurements of both the cell number and content of ATP, we developed a chimeric protein consists of CPPs and luciferase. An important feature of CPPs is that they can carry a variety of biological active substances into cells, including small molecule compounds, dyes, polypeptides, proteins, plasmid DNA, siRNA, liposomes, viruses and phage particles[22–26]. The advantage of CPPs as a carrier lies in its low toxicity without cell type restriction[27]. Most of these CPPs studies are TAT, a transcriptional activator of HIV-1 discovered in 1988[28]. Further studies have shown that heterologous proteins covalently bound to TAT can be transferred into cells[29]. In the present study, we demonstrated that the present TAT-LUC can sense cell viability precisely by penetrating the cell membrane without affecting its integrity or interfering with cellular metabolism (Fig 1B).

## Materials and methods

### Construction of pET-28a-TAT-LUC plasmid vector

Primers were designed based on the plasmid containing firefly luciferase sequence. Synthetic oligonucleotides were purchased from Genewiz (Suzhou, China). The gene coding for TAT-LUC was obtained from Bluelife Biotechnology (Guangzhou, China) that constructed by linking TAT gene with LUC gene using ligation-mediated polymerase chain reaction (PCR). TAT-LUC was then amplified using forward primer (5'-CGCGGATCCATGAGGAAGAAGCGGAGACAGCGACGAAGAGAAGACGCCAAAAACATAAA-3') containing a *Bam*H I site and the reverse primer (5'-CCGCTCGAGCACGGCGATCTTTCCGCCCTTCTTGGC-3') containing an *Xho* I site. The PCR was performed using KOD plus neo DNA Polymerase (ToYoBo, Shanghai, China) with the following cycle parameters: initial denaturation temperature of 94°C for 3 min, followed by 35 cycles of 98°C for 15 s, 58°C for 15 s and 68°C for 30 s followed by 68°C for 10 min, stored at 16°C. The PCR amplified products were purified by 1% agarose gel electrophoresis. The TAT-LUC PCR product was digested with *Bam*H I and *Xho* I, and the product was purified using 1% agarose gel electrophoresis. It was then ligated to *Bam*H I and *Xho* I digested pET-28a vector to generate the recombinant construct pET-28a-TAT-LUC (Fig 1C). The recombinant plasmid was transformed into competent *E*. *coli* DH5α and sequenced to confirm nucleotide identity. Then spread onto agar plate containing kanamycin (50 μg/mL) to allow selection of colonies that successfully incorporated the plasmids. Plasmid DNA extraction was performed using the High-purity plasmid small extraction kit (Tiangen-Biotech, Beijing, China). The extracted plasmids were identified by restriction enzyme digestion. The digested products were separated on a 1% agarose gel containing ethidium bromide. Nucleotide sequencing was carried out in the Sangon Biotech (Shanghai, China).

### Expression of TAT-LUC

The recombinant protein TAT-LUC was induced by IPTG and the overexpressed protein was isolated and analyzed by 10% polyacrylamide SDS-PAGE. In brief, the pET-28a-TAT-LUC plasmid vector was transformed into *E*. *coli* BL21 (DE3) cells and a single colony was picked from the kanamycin (50 μg/mL) Luria-Bertani (LB) agar plate after one day culture. It was inoculated in 5 mL LB broth supplemented with 50 μg/mL kanamycin. The culture was incubated at 37°C with continuous shaking at 210 rpm on shaking incubator overnight. 5 mL of this primary culture was inoculated in 500 mL culture, and incubated at 37°C with shaking until the OD600 reached about 0.5–0.6. The cells were cooled to 22°C and IPTG was added to a final concentration of 0.5 mM, followed by 16 h of culture at 22°C. The bacterial was harvested by centrifugation (5000 rpm for 10 min at 4°C) and the cell pellets were resuspended in 20 mL of buffer A (20 mM Tris-HCL, pH 8.0, 500 mM NaCl and 10% glycerin). Uninduced and induced bacterial cells were disrupted by sonication, and the supernatant was collected by centrifugation (10000 rpm for 20 min at 4°C). An uninduced culture containing only the recombinant plasmid served as the control. Whole bacterial proteins, supernatant and pellet were analyzed by 10% polyacrylamide SDS-PAGE.

### Purification of TAT-LUC

A Ni-NTA resin column (7 sea-biotech, China) was used to purify TAT-LUC protein. The collected supernatant was filtered through a 0.45 μM membrane. The Ni-NTA affinity column was equilibrated with 10 column volumes of buffer A, and the filtrate was applied to the purification column. The protein was naturally bound to the column under gravity and repeated 1–2 times. The column was then washed with 10 volumes of buffer A. Twenty volumes of wash buffer (20 mM Tris-HCl, pH 8.0, 20 mM imidazole, 500 mM NaCl and 10% glycerin) were used to wash the column. Target protein was eluted by the addition of five volumes of elution buffer (20 mM Tris-HCl, pH 8.0, 200 mM imidazole, 500 mM NaCl and 10% glycerin). The eluate was collected and stored at 4°C. The purification procedure was performed at 4°C. The eluate was identified by SDS-PAGE.

The purified protein was loaded into 10 KDa molecular weight dialysis bag (Thermo Fisher Scientific, America) and dialyzed in dialysis buffer (20 mM Tris-HCl, pH 8.0, 10 mM EDTA,5 mM MgCl_2_ and 10% glycerin) overnight with magnetic stirring at 4°C. Protein concentration after dialysis was determined using the Bicinchoninic acid protein assay (Tiangen-Biotech, Beijing, China). The enzyme activity of the recombinant protein was determined by adding ATP, Mg^2+^ and substrate D-luciferin (sodium salt, Macklin, Shanghai, China).

### Tumor cell culture

Tumor cell lines (MDA-MB-231, Huh-7) were obtained from the Guangzhou Institutes of Biomedicine and Health and drug-resistant tumor cell lines (Skov-3/DDP, A549/DDP) were obtained from the Guangdong Provincial Hospital of Chinese Medicine. Cells were cultured in RPMI-1640 medium supplemented with 10% fetal bovine serum (FBS), 100 U/mL penicillin, and 100 U/mL streptomycin under 5% CO_2_ at 37°C.

### Determination of TAT-LUC enzyme activity

For the cell-free luciferase assay, luciferin substrate solution was prepared containing 50 mg D-luciferin in 5 mM MgCl_2_ using ultra-pure ATP-free water. ATP (Dual-Luciferase Reporter Assay System; Promega) was diluted in ATP-free water with a final concentration of 10 μM, 5 μM, 2.5 μM, 1.25 μM, 625 nM, 313 nM, 156 nM, 78 nM, 39 nM, 20 nM, 10 nM, 0 nM. 1 μL of TAT-LUC (3.1 mg/mL) and 1 μL of D-luciferin (15 mg/mL) were added to 100 μL of different concentrations of ATP solution. Luciferase activity was acquired by using a GloMax^®^ 96 Microplate Luminometer (Promega, America) immediately. In addition, the luciferase (LUC) in the ATP assay kit (Beyotime, China) was used as a control.

For intracellular ATP assay, the best incubation time was tested firstly using a kinetic study. Briefly, different numbers of Skov-3/DDP cells (0–20,000 cells) were cultured in black-walled 96-well microtiter plates (Croning, USA). The culture medium was replaced with 100 μL ATP-free fresh medium and TAT-LUC or LUC was added to reach a final concentration of 3.1 mg/mL. The culture medium was replaced by D-luciferin containing fresh medium (150 μg/mL) at different time course. The luminescence was measured by using a GloMax^®^ 96 Microplate Luminometer. The optimal time for TAT-LUC to enter cells was determined by correlation analysis of cell number and luminescence intensity. In addition, cell lysis based luciferase assay was used as a control. The culture medium of Skov-3/DDP adherent cells (0–20000 cells) in the culture plate was removed, and 40 μL of cell lysis solution (ATP assay kit, Beyotime, China) was added to each well. After lysis, the supernatant was collected by centrifugation (12000 rpm for 5 min at 4°C). Then, the supernatant was added to a black microplate containing ATP detection solution, the luminescence was measured immediately.

### Detection of chemosensitivity of tumor cells based on TAT-LUC

Tumor cells were tested for their sensitivity to paclitaxel (PTX; Aladdin, Shanghai, China), cisplatin (DDP; Aladdin, Shanghai, China) carboplatin (CBP; Aladdin, Shanghai, China), gemcitabine (GEM; Aladdin, Shanghai, China), vinorelbine (NVB; Aladdin, Shanghai, China), doxorubicin (DOX; Takara Biotechnology Co., Ltd., Dalian, China), mitomycin (MMC; Jiangsu Hengrui Medicine Co., Ltd., Jiangsu, China), 5-fluorouracil (5-FU; Takara Biotechnology Co., Ltd., Dalian, China) and vincristine (VCR; Aladdin, Shanghai, China) using this assay.

Chemodrug concentrations were used at six different doses of the peak plasma concentration (PPC, 6.25%, 12.5%, 25%, 50%, 100%, 200%). The PPCs were based on the clinical dosage that adjusted to give good discrimination (Table 1). Three parallel repeats were used for each concentration. M_0_ wells were set as drug-free controls. Cell suspension was adjusted to 0.5–1×10^5^ cells/mL and 100 μL of the cell suspensions were seeded to each well of a 96-well black microplate. The plates were incubated in a cell incubator at 37°C in a 5% CO_2_ for 1–2 days. Then, the culture medium was replaced with the ATP-free fresh medium containing TAT-LUC (3.1 mg/mL). After TAT-LUC penetrating into cells, the culture medium was replaced by D-luciferin (150 μg/mL) containing fresh medium to analyze the cell viability as described above.

**Table 1:** Drugs used in the study and their 100% PPC.

Drug	100% PPC(μg/mL)
Paclitaxel (PTX)	10
Cisplatin (DDP)	2.5
Carboplatin (CBP)	25
Gemcitabine (GEM)	25
Vinorelbine (NVB)	10
Doxorubicin (DOX)	1
5-Fluorouracil (5-FU)	25
Mitomycin (MMC)	0.5
Vincristine (VCR)	0.4

### MTT assay

Cell viability, as a testing endpoint of chemodrug cytotoxicity, was determined with MTT cell proliferation and cytotoxicity assay kit (BestBio, Shanghai). Briefly, 100 μL of tumor cells were seeded into 96-well plates at a density of 0.5–1×10^4^ cells per well and incubated with six different doses of the PPC of chemodrug for 1–2 days. At the end of the culture, 10 μL of MTT (3-(4,5-dimethyl-2-thiazolyl)-2,5-diphenyltetrazolium bromide) solution was added into each well and the plate was incubated for 4 hours at 37°C. After removing incubation medium, the formazan crystals were dissolved in 150 μL solution of DMSO. The absorbance was measured at 570 nm using a spectrophotometer (Biotek Epoch, America). Three parallel repeats were used for each concentration. C wells were set as drug-free controls. Z wells were set as zero controls, which only contained the medium, MTT and DMSO.

### Data analysis

Data were interpreted and compared using three parameters. Sensitivity index (SI) is calculated as the 600 minus the sum of the percentage inhibition at each concentration tested [SI = 600-sum (Inhibition at 200, 100, 50, 25, 12.5 and 6.25% PPC)] [12]. In the TAT-LUC based assay, inhibition rate of tumor cells proliferation (IR) = (1.0-Test/M_0_) ×100%. In MTT assay, IR = (C-Test)/ (C-Z) ×100%. Four categories of *in vitro* sensitivity were defined as: (a) strong sensitivity, IC90≤100% PPC and IC50<25% PPC; (b) partial sensitivity, IC90>100% PPC and IC50≤25% PPC; (c) weak sensitivity, IC90≤100% PPC and IC50>25% PPC or SI≤250; and (d) resistance, IC90>100% PPC and IC50>25% PPC and SI>250[30, 31].

Dose-response curve fitting and data analysis were performed using linear regression with Origin Pro (version 17.0, Hampton, Massachusetts, USA). Each result is represented as mean ± SD. To detect statistical correlations, the Spearman’s rank correlation coefficient (R^2)^ was calculated between the luciferase activity and ATP concentration or cell number.

## Results and discussion

### Cloning and identification of TAT-LUC

**Fig 2:**
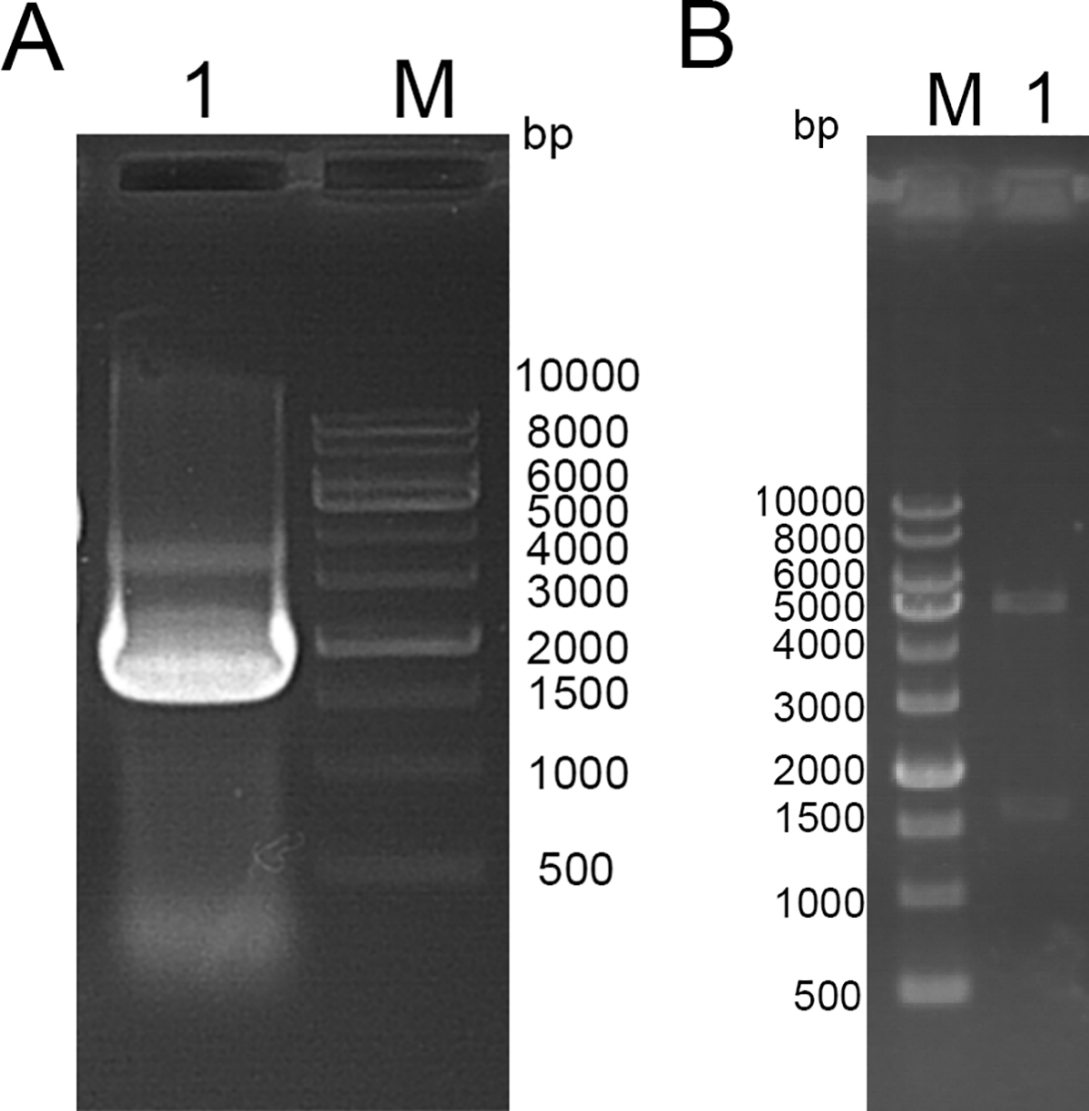
Identification of TAT-LUC. (A) Lane 1: PCR amplification products; Lane M: 1 kb DNA marker. (B) Double digestion of recombinant plasmids pET-28a-TAT-LUC with *Xho* I and *Bam*H I. Lane M: 1 kb DNA marker; lane 1: TAT-LUC plasmid digested products.

The constructed TAT-LUC is consistent with the forecast amplification band close to the 1500 bp Marker (Fig 2A). The TAT-LUC was cloned into pET-28a and transformed into in *E*. *coli* DH5α host cells. The recombinant plasmids were extracted and identified by restriction enzyme digestion. The resulting product corresponded to a 1600 bp band in the gel (Fig 2B). Sequencing showed that the TAT-LUC gene of 1677 bp was successfully cloned into the vector pET-28a (S1 Fig).

### Recombinant TAT-LUC expression and purification

**Fig 3:**
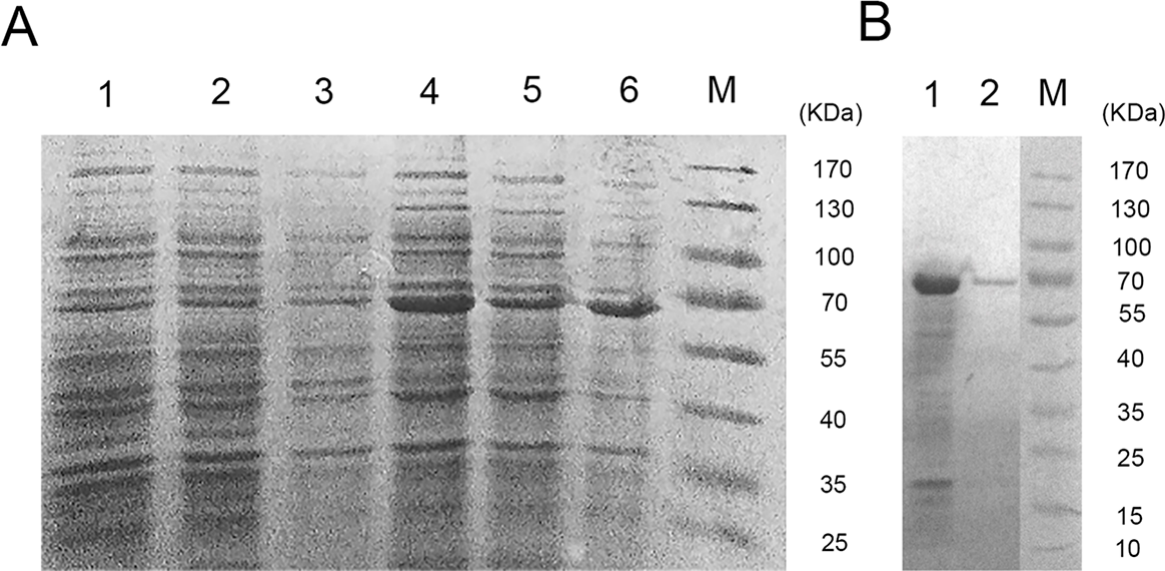
SDS-PAGE of TAT-LUC protein. (A) The protein was induced with IPTG (0.5 mM) at 22°C for 16 h in E. coli BL21(DE3) cells, and analyzed by 10% polyacrylamide SDS-PAGE. Lane 1, uninduced whole bacterial proteins; lane 2, supernatant of uninduced cell lysate; lane 3, pellet of uninduced cell lysate; lane 4, induced whole bacterial proteins; lane 5, supernatant of induced cell lysate; lane 6, pellet of induced cell lysate; Lane M, molecular weight marker. (B) Isolation of TAT-LUC. Lane 1, supernatant of cell lysate; Lane 2, purified TAT-LUC; lane M, molecular weight marker.

Recombinant pET-28a-TAT-LUC was successfully expressed heterologously in *E*. *coli* BL21(DE3) and purified by Ni-NTA affinity chromatography. The expression and purification of TAT-LUC were analyzed by SDS-PAGE. Compared with the uninduced group (Fig 3A, lanes 1–3), the recombinant protein was expressed in both the supernatant and precipitate in induced group (Fig 3A, lanes 4–6), and the apparent molecular weight of TAT-LUC was approximately 68 kDa. After Ni-NTA affinity purification, TAT-LUC showed a single band with a molecular weight of approximate 68 KDa as expected (Fig 3B, lane 2).

### Activity of TAT-LUC

**Fig 4:**
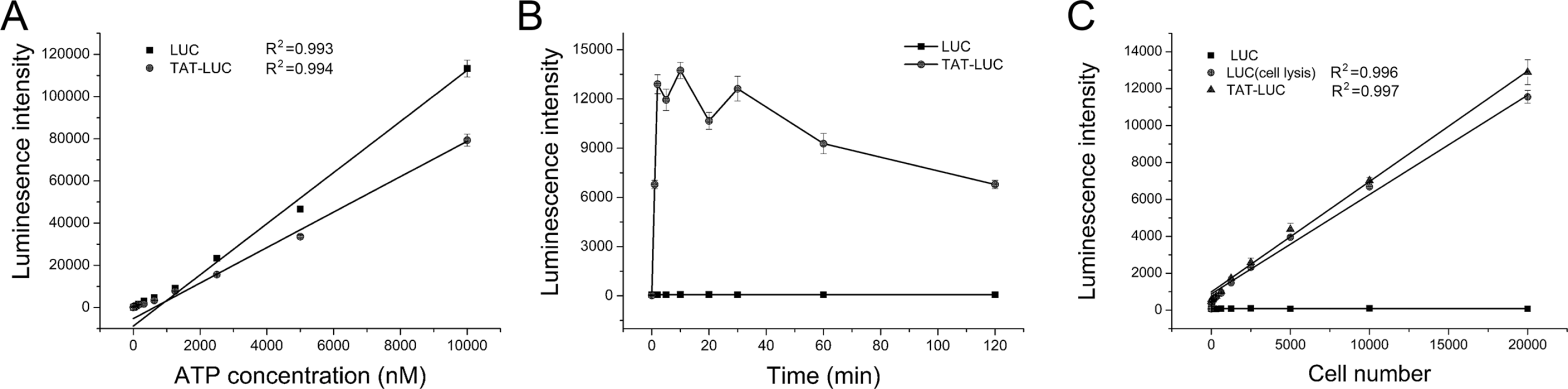
The activity of TAT-LUC or LUC for ATP and living cell detection. (A) TAT-LUC and LUC for free ATP detection. The correlation between the luminescence intensity (y-axis) and ATP concentration (μM, x-axis) is plotted. Linear fitting equation of TAT-LUC: Y = -5217.44709+8.40631X, Linear fitting equation of LUC: Y = -875098299+12.11998X. (B) The time of tumor cell uptake of TAT-LUC and LUC. (C) TAT-LUC, LUC for intracellular ATP detection and cell lysis based luciferase assay for ATP detection. Linear fitting equation of TAT-LUC: Y = 986.30052+0.59832X, Linear fitting equation of LUC (cell lysis): Y = 880.32237+0.53831X.

Compared with LUC (R^2^ = 0.993), activity of TAT-LUC was also proportional to the ATP concentration in cell-free luciferase assay (Fig 4A, R^2^ = 0.994). Although TAT-LUC detected a relatively low luminescence intensity during the entire concentration range of ATP, the sensitivity of TAT-LUC was still capable of detecting 10 nM ATP. The time-course of transduction showed that the TAT-LUC was rapidly transduced into living tumor cells within a few minutes, while control LUC was unable to detect the intracellular ATP (Fig 4B). The cellular uptake of TAT-LUC reached a maximum at 2 min, and then stabilized over 30 min. Thus, 2 min was the incubation time. The feasibility of this assay for *in vitro* test was conducted using Skov-3/DDP cells. Skov-3/DDP cells were serially diluted to give from 0 to 20,000 cells per well and the luminescence was measured 2 min after the addition of TAT-LUC and luciferin. As few as 40 cells were detected by this assay. With the same method, TAT-LUC had the same sensitivity for detecting other cell types (A549/DDP, MDA-MB-231, Huh7). A strong correlation was found between the intensity of luminescence signal and cell number over a wide range of cell numbers (Fig 4C, R^2^ = 0.997). In comparison, the luminescence intensity measured with LUC treatment was significantly lower than that of TAT-LUC, which explained TAT-LUC had the ability to transduce into cells. The comparison between TAT-LUC assay and cell lysis based luciferase assay was conducted in our pre-experiment. The classical luciferin-luciferase assay was able to detect 100 cells (Fig 4C, R^2^ = 0.996). Besides, cell lysis based luciferase assay showed that LUC detected a relatively low luminescence intensity during the entire concentration range of cell number in comparison with TAT-LUC. This indicated that ATP was partially lost during the extraction of ATP by cell lysis. The results demonstrated that TAT-LUC was a reliable and sensitivity tool for the measurement of intracellular ATP in living cells.

### Chemosensitivity analysis of tumor cells

Previous experiments confirmed the feasibility of TAT-LUC in the detection of intracellular ATP. The TAT-LUC based assay and MTT assay were then used for the analysis of chemosensitivity of different tumor cells. Four strains of tumor cell (Skov-3/DDP, A549/DDP, MDA-MB-231, Huh-7) were tested. Tumor cells were incubated with different concentrations of chemodrugs for 1–2 days. After cell culture in the TAT-LUC based assay, the culture medium was replaced with the ATP-free fresh medium containing TAT-LUC. TAT-LUC was incubated for 2 min, and the luminescence signal was recorded after culture medium was replaced by D-luciferin containing fresh medium.

**Fig 5:**
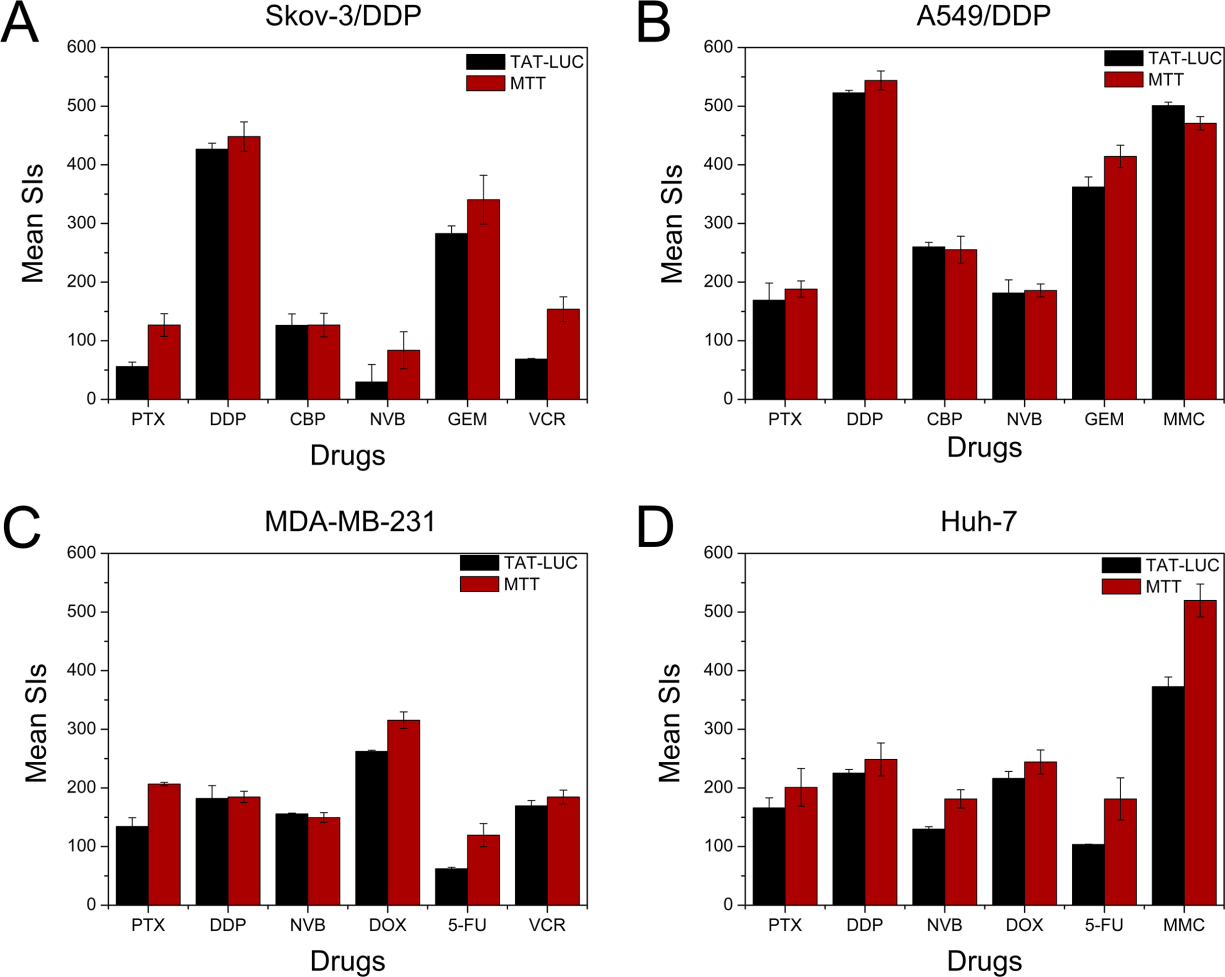
Comparison of the TAT-LUC based assay (black bars) and MTT assay (red bars) of tumor cell sensitivity indices (SIs). The suitable cut off point of SI is 250. Data represent means ± SD from three repeats. (A) Skov-3/DDP, (B) A549/DDP, (C) MDA-MB-231, (D) Huh-7. PTX, paclitaxel; DDP, cisplatin; CBP, carboplatin; GEM, gemcitabine; NVB, vinorelbine; DOX, doxorubicin; 5-FU, 5-fluorouracil; MMC, mitomycin; VCR, vincristine.

Sensitivity index is a natural logarithmic index, ranging from 0–600 for inhibition. SI of zero corresponding to complete cell kill and 600 corresponding to no effect, with the suitable cut-off point of 250[31, 32]. The MTT assay is a commonly used method for *in vitro* tumor chemosensitivity testing. In order to evaluate the TAT-LUC based assay, the sensitivity index measured by TAT-LUC based assay and MTT assay were compared. The TAT-LUC based assay was able to measure the ATP released from less than 40 cells, while the MTT assay could not detect less than 500 cells/well. The sensitivity index measured by TAT-LUC based assay was generally lower than that of MTT assay, which reflects the inhibition rate of TAT-LUC based assay was higher than MTT assay (Fig 5). The drug sensitivity of the two methods was almost identical, and the TAT-LUC based assay was more sensitive than the MTT assay.

**Fig 6:**
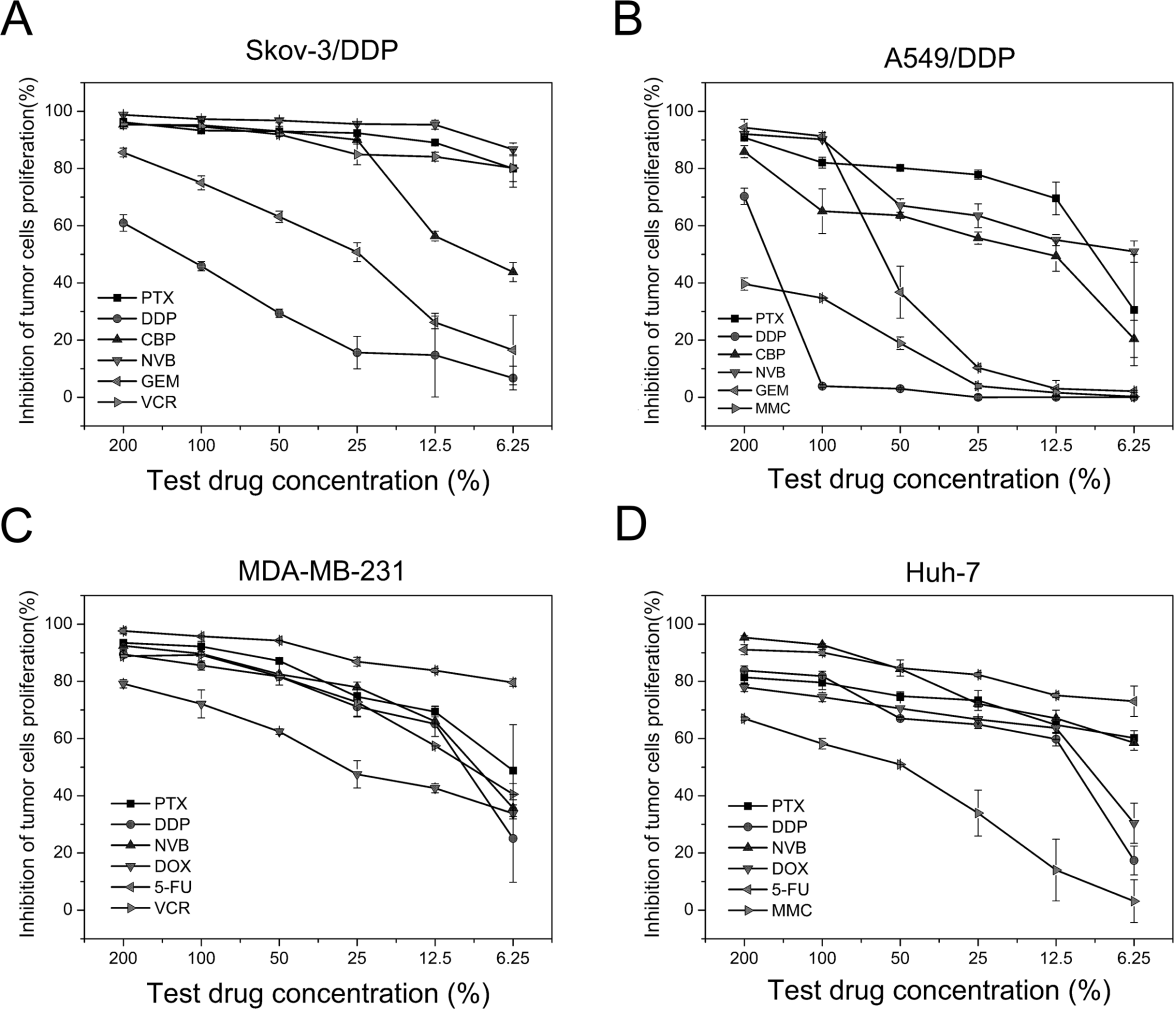
Chemotherapy dose-inhibition curve in the TAT-LUC based assay. The X axis is the test drug concentration percentage, and the Y axis is the inhibition rate of tumor cells proliferation. (A) Chemosensitivity of Skov-3/DDP. (B) Chemosensitivity of A549/DDP. (C) Chemosensitivity of MDA-MB-231. (D) Chemosensitivity of Huh-7. PTX, paclitaxel; DDP, cisplatin; CBP, carboplatin; GEM, gemcitabine; NVB, vinorelbine; DOX, doxorubicin; 5-FU, 5-fluorouracil; MMC, mitomycin; VCR, vincristine.

In the TAT-LUC based assay, the chemosensitivity of Skov-3/DDP was in the following order: NVB>PTX>VCR>CBP>GEM>DDP (Fig 5A). The agent with the lowest SI values for Skov-3/DDP was NVB (mean SI, 29.58), followed by PTX (mean SI, 56.01), VCR (mean SI, 68.59), CBP (mean SI, 126.22) and GEM (mean SI, 282.77). The agent with the highest SI for Skov-3/DDP cell was DDP (mean SI, 426.65). Chemosensitivity of Skov-3/DDP tumor cells can be divided into the following two levels: 1) strong sensitive to NVB, PTX, VCR and CBP, 2) resistance to DDP and GEM (Fig 6A). The agent with the lowest SI values for A549/DDP was PTX (mean SI, 169.01), followed by NVB (mean SI, 181.25), CBP (mean SI, 259.95), GEM (mean SI, 362.11) and MMC (mean SI, 501.01) (Fig 5B). The agent with the highest SI was DDP (mean SI, 522.689). Chemosensitivity of A549/DDP can be divided into the following 3 levels: 1) strong sensitive to NVB, 2) partial sensitive to PTX, MMC, CBP and DDP, 3) resistant to GEM (Fig 6B). The agent with the lowest SI values for MDA-MB-231 was 5-FU (mean SI, 62.1), followed by PTX (mean SI, 134.34), NVB (mean SI, 155.73), VCR (mean SI, 169.45) and DDP (mean SI, 181.98). The agent with the highest SI was DOX (mean SI, 262.33) (Fig 5C). Chemosensitivities of MDA-MB-231 can be divided into the following 3 levels: 1) strong sensitive to 5-FU and PTX, 2) partial sensitive to NVB, VCR and DDP, 3) resistant to DOX (Fig 6C). The agent with the lowest SI values for Huh-7 was 5-FU (mean SI, 103.65), followed by NVB (mean SI, 129.76), PTX (mean SI, 165.81), DOX (mean SI, 216.28) and DDP (mean SI, 225.2). The agent with the highest SI was MMC (mean SI, 372.76) (Fig 5D). Chemosensitivities of Huh-7 can be divided into the following 3 levels: 1) strong sensitive to 5-FU, 2) partial sensitive to NVB, PTX, DOX and DDP, 3) resistant to MMC (Fig 6D).

In the chemosensitivity assay, we performed MTT assays to verify that the TAT-LUC based assay was reliable and sensitive. These results indicated that TAT-LUC was able to detect the ATP content of tumor cells successfully, and the sensitivity of the drug could be determined by calculating the inhibition of tumor cells proliferation at each concentrations of each drug.

## Conclusion

The aim of this study is to develop a simple method for measuring the intracellular ATP in living cells. To achieve this goal, we created a fusion protein, TAT tagged luciferase. Compared with LUC, TAT-LUC was capable of penetrating the cells. The TAT-LUC based assay had a good sensitivity, which could detect as low as 10 nM ATP or 40 tumor cells. TAT-LUC was further used for the analysis of drug sensitivity of four types of tumor cells (Skov-3/DDP, A549/DDP, MDA-MB-231, Huh-7), which was proven to be reliable and sensitive in comparison with MTT assay. The greatest advantage of TAT-LUC based assay is that it allows the rapid and direct measurement of the intracellular ATP content in living tumor cells without lysing. It avoids the ATP degradation and potential operational error. Therefore, single or very little tumor specimens can be used for drug sensitivity testing, greatly improving the detection speed. Furthermore, it enables real-time reflection of tumor drug resistance and accelerates the detection rate, which can be a valuable aid for personalized cancer chemotherapy.

## Supporting information

S1 Fig Sequencing results: TAT-LUC sequence. Red represents the TAT tag.
(DOCX)
