# Supplementary figures and images for: A simple in vitro tumor chemosensitivity assay based on cell penetrating peptide tagged luciferase
Source: PLoS One. 2017 Nov 10;12(11):e0186184. doi: 10.1371/journal.pone.0186184 (PMC5681261; doi:10.1371/journal.pone.0186184)

**S1 Fig. Sequencing results: TAT-LUC sequence. Red represents the TAT tag.**


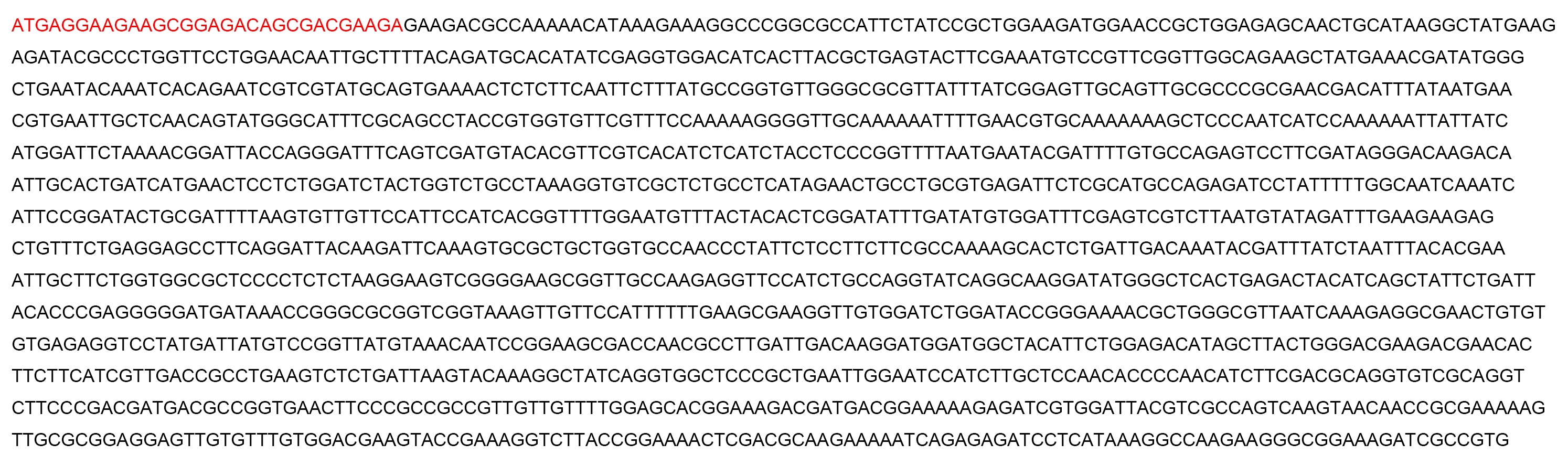

Supplement: S1 Fig — Red represents the TAT tag. (DOCX) [file pone.0186184.s001.docx]
